# A systematic analysis of the burden of disease attributable to occupational noise-induced hearing loss in China based on the 2019 global burden of disease study

**DOI:** 10.1186/s12889-024-21094-4

**Published:** 2024-12-18

**Authors:** Sirui Wang, Shuhan Liu, Kaijie Li, Wei Tang, Xiaofeng Fan, Yongran Cheng, Lili Dai

**Affiliations:** 1 Clinical Medicine Department, Hangzhou Normal University, Hangzhou, China; 2 Department of Otolaryngology, Hangzhou Xixi Hospital, Hangzhou, China; 3 School of public health, Hangzhou Medical college, Hangzhou, China; 4 Department of Otolaryngology, Affiliated Hospital of Hangzhou Normal University, Hangzhou, China

**Keywords:** Occupational noise, Hearing loss, China, Years lived with disability (YLDs), Global burden of disease study (GBD)

## Abstract

**Background:**

Occupational noise has long been recognized as a significant risk factor for hearing loss, particularly among workers. This study aimed to assess the disease burden of hearing loss caused by occupational noise in China from 1990 to 2019, with a focus on differences across sex and age groups, so as to address the existing gaps in the Global Burden of Disease Study (GBD) reports. By analyzing changes in years lived with disability (YLDs) among different sex and age groups over the past 30 years, this study provides valuable insights for the development of occupational noise safety measures in China.

**Methods:**

We extracted data on the burden of hearing loss attributable to occupational noise from the 2019 Global Burden of Disease study. R software (version 4.12) was used to calculate the YLDs, age-standardized rates (ASRs), and average annual percent change (AAPC), stratified by age and sex. The Joinpoint regression model was used to analyze the trends in the burden of disease attributable to occupational noise exposure from 1990 to 2019.

**Results:**

In China, the YLDs attributable to occupational noise-induced hearing loss in 2019 were 2.3277 million [95% uncertainty interval (UI): 1.5779–3.3478 million, marking a 70.95% increase compared with that in 1990. Throughout the study period, YLD rates exhibited a declining trend, with rates of 127.7 per 100,000 (95% UI: 87.4–181.4) in 1990 and 119.8 per 100,000 (95% UI: 81.2–182.1) in 2019. Using the Joinpoint regression model, the annual percent change (APC) in age-standardized YLD rates for occupational noise-related hearing loss initially increased from 1990 to 2000, followed by a decline, reaching its lowest point in 2015. From a sex perspective, the burden of YLDs in Chinese males exceeded that in females, although the rate of decline was less pronounced in females. With regard to age, the number and rate of YLDs attributable to occupational noise-induced hearing loss generally increased with age, particularly among middle-aged individuals and older adults. However, from 1990 to 2019, the number of YLDs attributable to occupational noise-induced hearing loss decreased among young people aged 15–19 years, accompanied by a significant reduction in YLDs rates.

**Conclusion:**

Hearing loss attributable to occupational noise represents a substantial public health concern, especially among middle-aged and older adult workers in China. These findings underscore the importance of implementing effective measures to mitigate occupational noise exposure.

## Introduction

Hearing loss, characterized by a hearing threshold of 20 dB or higher, can manifest as mild, moderate, severe, or profound, impacting one or both ears and causing difficulty in hearing conversational speech or loud sounds [1]. The effects of hearing loss are far-reaching and profound, encompassing loss of communication ability, delayed language development in children, leading to social isolation, loneliness, and depression, particularly among older adults [2]. Inadequate facilities for addressing hearing loss in many regions affect educational attainment and career prospects [3]. According to the GBD study, over 1.5 billion people, nearly 20% of the global population, currently endure hearing loss, with 430 million experiencing disabling hearing loss [4]. By 2050, this number is projected to surpass 700 million [5–7]. Data from the Second National Sample Survey on Disability in China in 2006 revealed approximately 27.8 million individuals with hearing and speech disabilities in China, including over 2 million deaf-mutes, with an annual increase of over 30,000 [8].

Among the myriad risk factors for hearing loss, occupational noise emerges as a significant contributor [9]. The diagnostic criteria for occupational noise deafness are based on several key indicators, including a history of occupational noise exposure for > 3 years, progressive hearing decline, the presence of tinnitus, and the diagnosis of sensorineural deafness, as confirmed through pure tone audiometry. Additionally, these criteria involve the integration of occupational health surveillance data and on-site occupational hygiene investigations, excluding other potential causes of auditory damage, and provide a comprehensive understanding of an individual’s occupational noise exposure and its impact on hearing health. It represents a chronic auditory injury evolving from physiological responses to pathological changes, becoming perceptible when subjective sensations impact the speech frequency range. Such hearing loss disrupts work, affecting mood; moreover, prolonged exposure can also induce health complications [10, 11].

China, as a prominent industrial nation, grapples with prevalent occupational noise hazards. These hazards pervade diverse industries and affect a substantial workforce. Previous studies indicate that the mining sector exhibits the highest prevalence of hearing loss, with detection rates of hearing disorders and high-frequency hearing loss at 29.96% and 10.11%, respectively. The fourth national economic census highlights approximately 70,000 mining entities in China, including around 5.96 million individuals, comprising 1.55% of the workforce in legal entities [12]. Noise levels in mining environments, except during machinery repair, often surpass occupational exposure limits, particularly for personnel engaged in rock drilling and advancement, where noise intensity exceeds 90 d [13]. While harmful noise is most prevalent in mining, manufacturing, and construction, workers across all industries face risks of hearing loss. For instance, personnel occupying roles such as gatekeepers or administrative staff in noisy workplaces are also susceptible to hearing impairment [14].

Despite the availability of hearing protection gear, many individuals still experience hearing loss [13]. Several studies have elucidated the role of occupational noise risk factors in the burden of disease attributed to hearing loss. Occupational noise-induced hearing loss ranks among the most frequently reported occupational diseases in Europe and other regions worldwide, contributing to 7–21% of hearing loss cases [15]. According to China’s Health and Health Affairs Development Statistical Bulletin for 2022, occupational noise-induced hearing loss ranks second only to pneumoconiosis, emerging as China’s second most significant occupational disease [16].

Although numerous studies on hearing loss across the globe have been attributed to occupational noise, comprehensive analyses specifically targeting China are not available. As a major industrial nation, China faces complex and widespread occupational noise exposure. This study aimed to assess the disease burden of hearing loss caused by occupational noise in China from 1990 to 2019, with a focus on differences across sex and age groups, so as to address the existing gaps in the GBD reports. By analyzing changes in YLDs among different sex and age groups over the past 30 years, this study provides valuable insights for the development of occupational noise safety measures in China.

## Methods

### Data sources and definitions

The GBD global burden of disease database uses comparative risk assessment to estimate attributable mortality rates, years of life lost(YLLs), years lived with disability (YLDs), and disability-adjusted life years (DALYs). It systematically investigates 87 risk factors across 204 countries and regions worldwide from 1990 to 2019 [17]. Information on age-related and other forms of hearing loss YLD numbers, YLD rates, and their age-standardized rates (ASR) in China from 1990 to 2019 was obtained from the Institute for Health Metrics and Evaluation (https://vizhub.healthdata.org/gbd-results/). DALYs consist of YLLs due to premature death and YLDs caused by disease. Hearing loss related to occupational noise typically does not lead directly to premature death, resulting in lower YLLs, with YLDs being the primary contributor. Therefore, we have chosen YLDs as the main measure of disease burden.

YLDs are calculated by multiplying the prevalence of a condition by its associated disability weight, which reflects the severity of the disease relative to all other health states [18]. Disability weights range from 0 (representing perfect health) to 1 (equivalent to death) [19].

Hearing loss is defined as a loss of hearing greater than 20 dB in the better ear, measured as the average of four frequencies (500 to 4000 Hz) [1]. Hearing loss is reported in the GBD by seven mutually exclusive severity categories [20].

### Statistical analysis

This study furnished data on YLDs numbers and ASRs of hearing loss attributed to occupational noise in China, stratified by age and sex, with 95% uncertainty intervals (UI). (UI) is used to represent the range of uncertainty around an estimated value. The 95% Uncertainty Interval (UI) means that, based on the existing data and model estimates, we have 95% confidence that the true value lies within this interval, with the remaining 5% probability outside of the interval. We computed 95% UIs based on 1000 draws from the posterior distribution of each stage in the estimation process using the 2·5th and 97·5th percentiles of the 1000 ordered values [21].

Regarding sex classification, participants are categorized into male, female, and total population. For age classification, participants are grouped into the following age ranges: 15–19 years, 20–24 years, 25–29 years, 30–34 years, 35–39 years, 40–44 years, 45–49 years, 50–54 years, 55–59 years, 60–64 years, 65–69 years, 70–74 years, 75–79 years, 80–84 years, 85–89 years, 90–94 years, and 95 years and above.

In this study, R software (version 4.12) was used to calculate the YLDs due to hearing loss caused by occupational noise exposure, followed by further stratified analyses (e.g., by sex and age group). Occupational noise is defined as the proportion of the population ever occupationally exposed to 85 + decibels of noise, based on population distributions across 17 economic activities [22]. Moreover, R software was employed to calculate the ASR and the AAPC in the trend analysis. The 95% confidence interval(CI) was also calculated. The trends were defined as upward (AAPC > 0) or downward (AAPC < 0).

Moreover, the study used the Joinpoint regression model to analyze trends in age-related and other forms of hearing loss YLDs standardized rates and YLDs from 1990 to 2019. This model, a time series trend analysis method, partitions the study timeframe into different intervals using multiple turning points. It constructs and refines regression models within each interval, facilitating a detailed assessment of the burden of disease trends and overall trends within each period [23].

The Joinpoint regression program (version 4.8.0.1) was used for analyzing the Joinpoint regression model, while R software (version 4.12) was utilized for all other data analyses. A p-value of < 0.05 was considered statistically significant.

## Results

### Burden of YLDs of hearing loss attributable to occupational noise in china during 1990–2019

From 1990 to 2019, China’s hearing loss YLDs numbers increased from 5.58 million person-years (95% UI: 3.74–7.97 million) to 10.48 million person-years (95% UI: 7.03–15.08 million), marking an 87.84% increase. Similarly, YLDs numbers attributable to occupational noise witnessed a significant upsurge, rising from 1.3616 million person-years (95% UI: 0.9234–1.9443 million) to 2.3277 million (95% UI: 1.5779–3.3478 million), a 70.95% increase.

In 1990, the number of YLDs attributable to occupational noise in China was 1.3616 million person-years (95% UI: 0.9234–1.9443 million), accounting for 24.42% of the total hearing loss YLDs. By 2019, this number had risen to 2.3277 million person-years (95% UI: 1.5779–3.3478 million), accounting for 22.22% of the total. Despite the increase in YLDs attributable to occupational noise exposure, their proportion within the total YLDs for hearing loss has shown a declining trend. Concurrently, the ASR YLDs attributable to occupational noise in China exhibited a decline from 1990 to 2019, with an AAPC of -0.21 (95%CI: -0.301 to -0.119), indicating an overall downward trend (Table 1).

**Table 1:** YLDs number, YLDs rate, and age-standardized mortality rate of occupational noise-induced hearing loss by sex in China in 1990 and 2019

Outcoms	Year	Sex	Number	95%UI		ASR	95%UI		Rate	95%UI	
				Lower	Upper		Lower	Upper		Lower	Upper
YLDs	1990	Both	1,361,597	923,373	1,944,303	127.7	87.4	181.4	115.0	78.0	164.3
		Female	579,745	393,982	819,893	110.7	75.9	156.6	101.1	68.7	143.0
		Male	781,852	529,498	1,124,544	144.6	98.4	206.4	128.1	86.8	184.3
	2019	Both	2,327,706	1,577,894	3,347,829	119.8	81.2	172.1	163.7	110.9	235.4
		Female	1,049,527	713,448	1,501,479	108.9	73.8	155.2	150.5	102.3	215.3
		Male	1,278,179	864,801	1,842,751	131.1	88.9	188.2	176.3	119.3	254.2

Abbreviations: YLDs, years lived with disability; ASR, Age-standardized rate(per 100,000 population); UI, Uncertainty interval

### Impact of age and sex on the burden of attributable to occupational noise-induced hearing loss

**Fig. 1:**
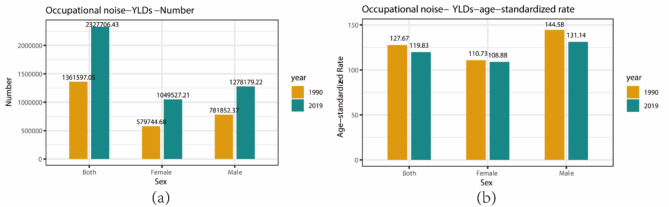
Comparison of YLDs number and age-standardized rate of occupational noise-induced hearing loss by sex in 1990 and 2019. (**a**) YLDs number; (**b**) YLDs age-standardized rate

The burden of hearing loss attributable to occupational noise varies significantly between sexes and age groups. In China, in 2019, the YLD numbers for hearing loss attributable to occupational noise were 1.28 million person-years for males (95% UI: 0.86–1.84) and 1.05 million for females (95% UI: 0.71–1.50 ), representing increases of 63.5% and 81.1% respectively since 1990 (Fig. 1a). The ASR YLDs for males in 2019 was 1.20 times that of females (Fig. 1b).

**Fig. 2:**
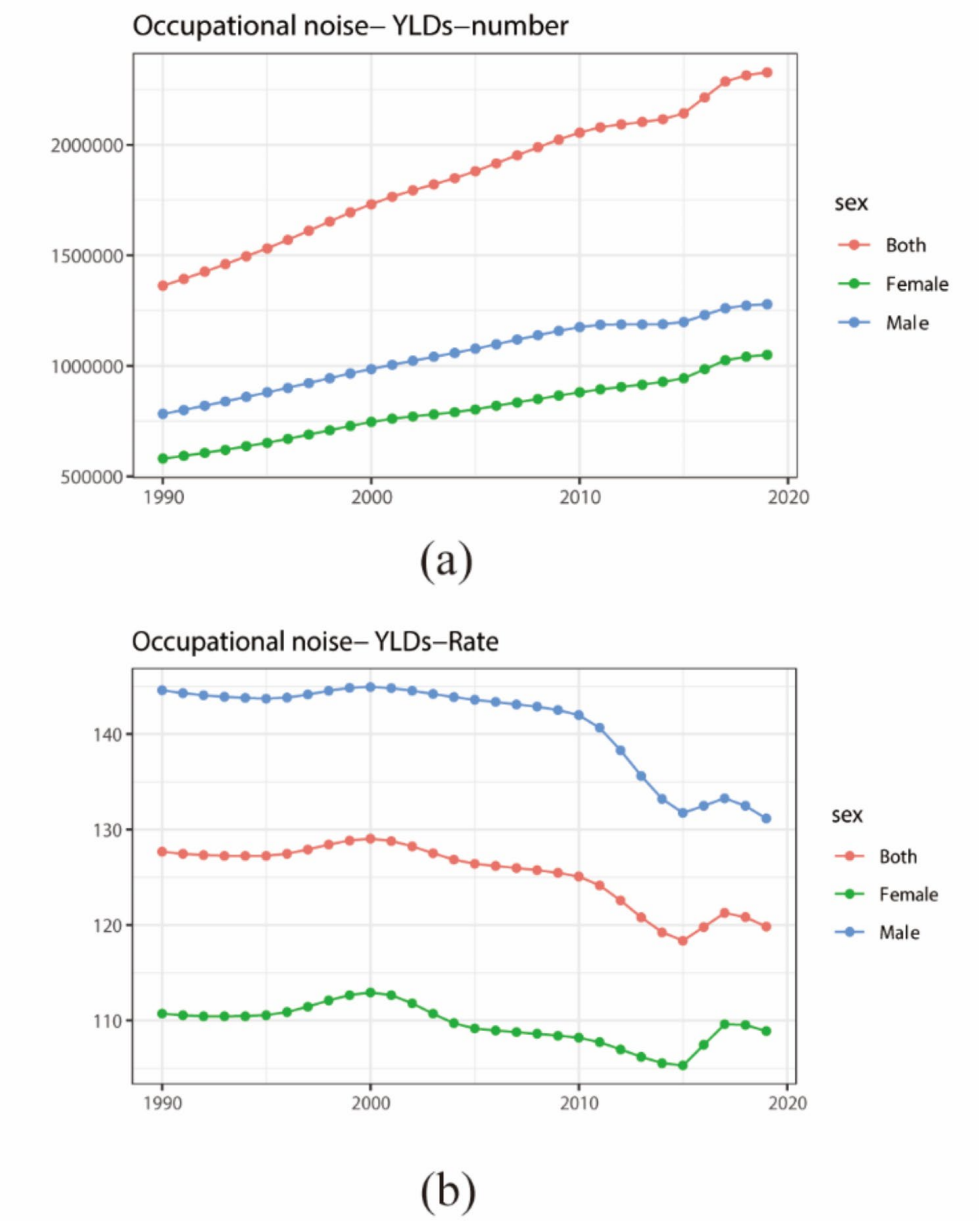
YLDs number and age-standardized rate of occupational noise-induced hearing loss by sex in 1990–2019. (**a**) YLDs number; (**b**) YLDs age-standardized rate

From 1990 to 2019, both males and females experienced an upward trend in YLD numbers, peaking in 2000 and subsequently declining, reaching the lowest point in 2015. Throughout this period, the ASR YLDs for males consistently exceeded those for females (Fig. 2).

**Fig. 3:**
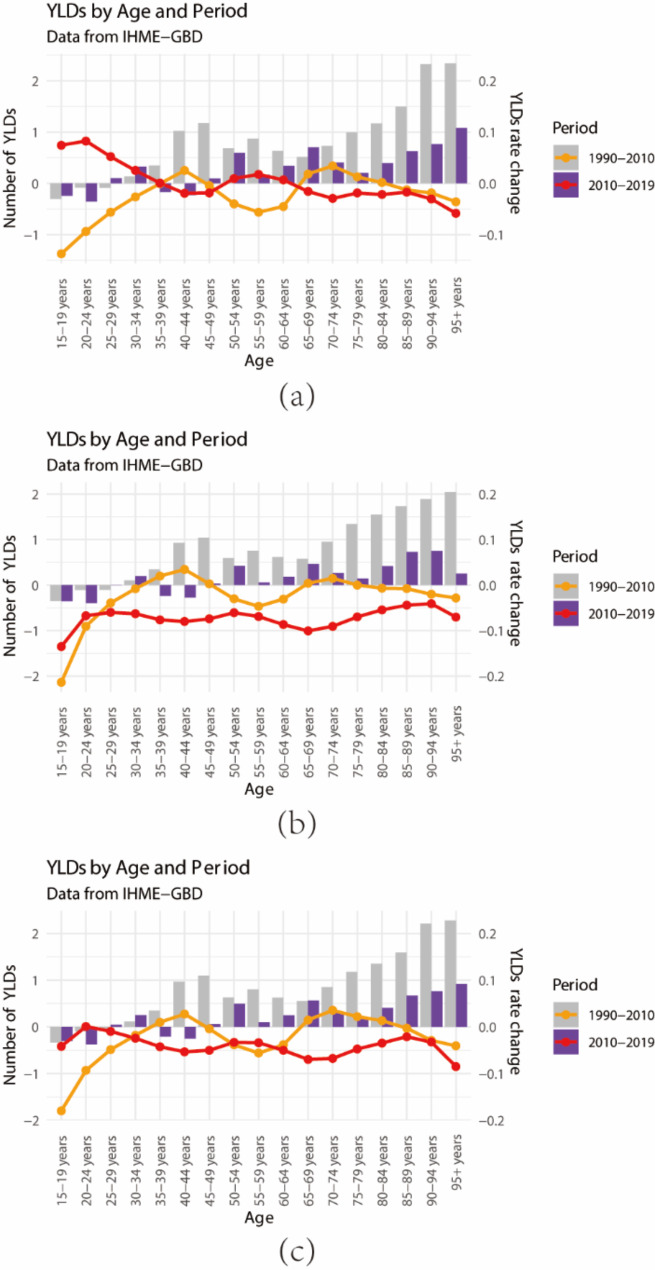
Annual percentage change in YLDs number and the rate of occupational noise-induced hearing loss by sex in different age groups during 1990–2010 and 2010–2019.The bar charts in each graph represent the number of YLDs for different age groups across two different time periods, with gray indicating the 1990–2010 period and purple representing the 2010–2019 period. The lines indicate the trend of YLDs rate changes for each age group, with the red line representing the 1990–2010 period and the yellow line representing the 2010–2019 period. (**a**) Female; (**b**) Male; (**c**) All

Between 1990 and 2010, the YLD numbers for the age groups of 15–19, 20–24, and 25–29 years exhibited a negative change, indicating a reduction in YLDs attributed to occupational noise among young individuals. Conversely, for those aged 65 years and over, the change values increased with age, indicating a rise in YLD numbers. Among the age groups of 35–39, 40–44, 65–69, 70–74, 75–79, and 80–84, the YLD rates increased in 2010 compared with those in 1990, while other age groups witnessed a decrease. From 2010 to 2019, a similar trend was observed, with YLD numbers for hearing loss among young individuals decreasing, albeit with a lesser increase in YLD numbers for those aged 65 years and over compared with the earlier period. Most age groups exhibited a decrease in YLD rates (Fig. 3a). The age groups for which both male and female YLD numbers decreased from 1990 to 2010 and from 2010 to 2019 were similar, specifically for the age groups of 15–19, 20–24, 35–39, and 40–44 years. In most age groups for males, the change values were negative, indicating a decrease in YLD rates, except for the age groups of 35–44 and 65–74 years during 1990–2010 (Fig. 3b). Conversely, in most age groups for females, including the age groups of 35–40, 40–44, 65–69, 70–74, 75–79, and 80–84 years (1990–2010) and 15–19, 20–24, 25–29, 30–34, 50–54, 55–59, and 60–64 years (2010–2019), the change values were positive, representing an increase in YLD rates (Fig. 3c).

**Fig. 4:**
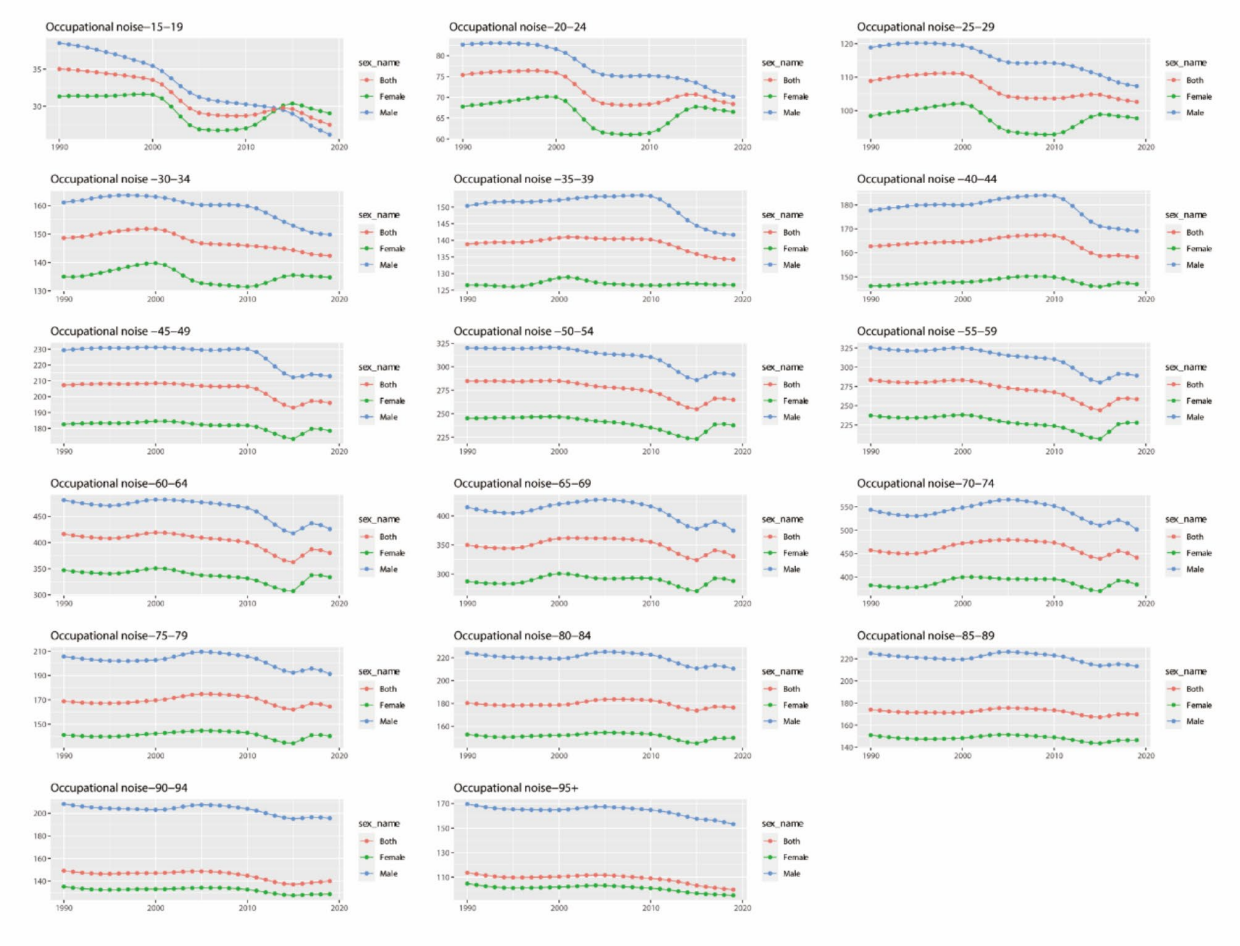
YLDs rate of occupational noise-induced hearing loss attributed to different age groups in China from 1990 to 2019. The figure is divided into multiple panels representing different age groups: 15–19 years, 20–24 years, 25–29 years, 30–34 years, 35–39 years, 40–44 years, 45–49 years, 50–54 years, 55–59 years, 60–64 years, 65–69 years, 70–74 years, 75–79 years, 80–84 years, 85–89 years, 90–94 years, and 95 years and above. The X-axis represents the years from 1990 to 2019, while the Y-axis indicates the YLDs rate for each age group, reflecting the burden of occupational noise-induced hearing loss. The lines in the graph represent different sex groups: blue for the total population (Both), red for females (Female), and green for males (Male)

From the age groups of 15–19 to 70–74 years, the YLD rates attributable to occupational noise mostly increased with age (Fig. 4). From 2010 to 2015, most male age groups exhibited a significant downward trend in the YLD rates, whereas females in the age groups of 15–19, 20–24, 25–29, and 30–34 years showed an upward trend (Fig. 4). Between 2000 and 2005, males in the age groups of 15–19, 20–24, 25–29, and 30–34 years experienced a notable decrease in YLD rates.

**Fig. 5:**
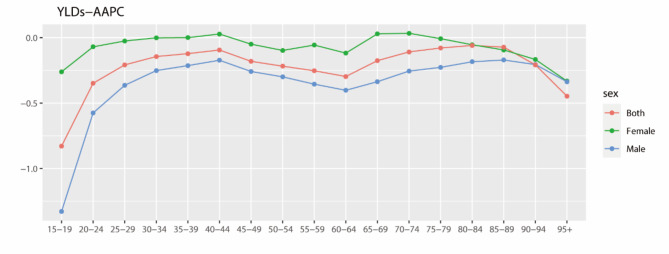
AAPC of YLDs rate attributed to occupational noise-induced hearing loss in China from 1990 to 2019

Age-period cohort analysis revealed the AAPC for YLD rates attributable to occupational noise for different age groups, showing similar downward trends for both males and females, with males mostly experiencing a greater decline (Fig. 5). However, for females, the age groups of 35–39, 40–44, 65–69, and 70–74 years all exhibited positive change values. Notably, the highest change value was observed for the age group of 70–74 years (3.38%), followed by the age group of 65–69 (2.94%) and 40–44 years (2.83%), indicating a bimodal pattern. In contrast, all male age groups showed negative change values.

For both males and females, the AAPC was lowest for the age group of 15–19 years, at − 132.9% and − 26.1% respectively, indicating the fastest decrease in hearing loss attributable to occupational noise in this age group. In contrast, in the age group of 20–24 years and older, the AAPC quickly increased to near zero, showing a relatively stable impact of occupational noise in these age groups.

### Joinpoint regression analysis of the burden of disease attributable to occupational noise-induced hearing loss in China

**Fig. 6:**
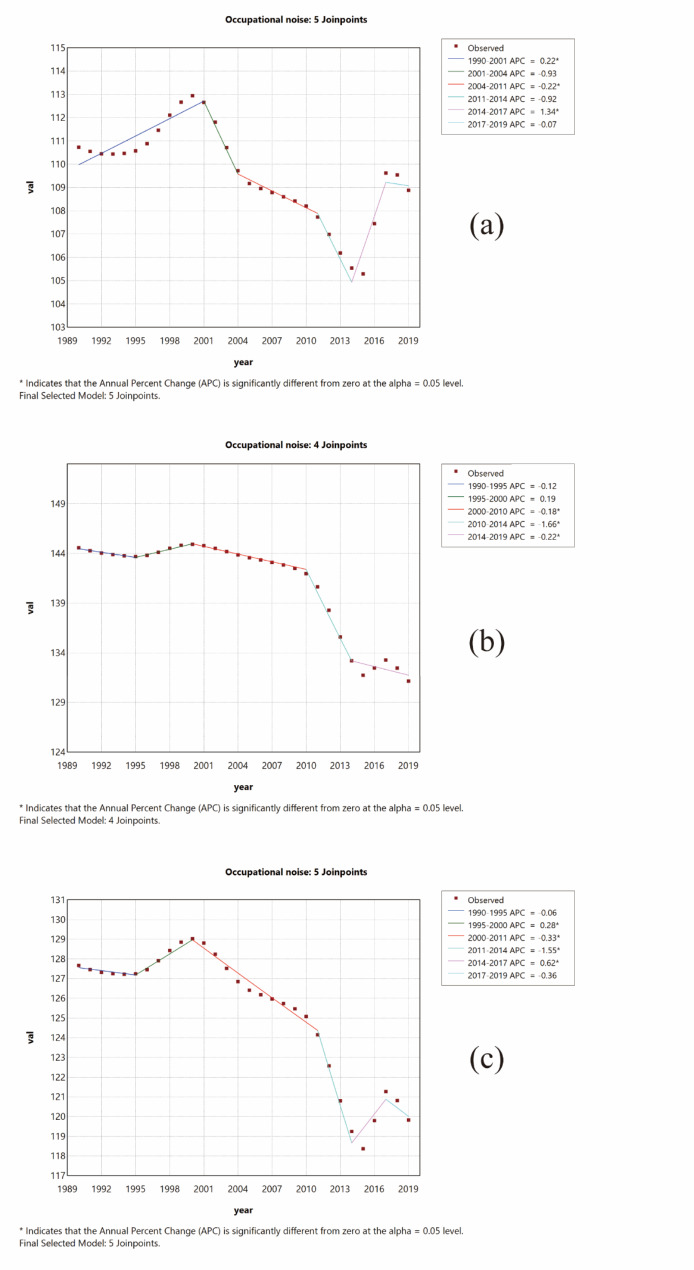
APC of Age-standardized YLDs rate attributed to occupational noise-induced hearing loss in China from 1999 to 2019 (*p < 0.05). (**a**) Female; (**b**) Male; (**c**) All

The Joinpoint regression analysis of YLDs attributable to occupational noise-induced hearing loss in China during 1999–2019 is depicted in Fig. 6. From 1990 to 1995, the annual percentage change (APC) in the age-standardized YLD rate (ASYLDR) for hearing loss attributable to occupational noise in China slightly decreased. Subsequently, from 1995 to 2000, it showed an increasing trend. From 2000 to 2014, a significant downward trend was observed. However, from 2014 to 2017, there was an upward trend, followed by a decrease after 2017, indicating a fluctuating state.

During 1990–2001, the APC in the ASYLDR for women in China showed an upward trend, followed by a significant downward trend from 2001 to 2014. However, from 2014 to 2017, an upward trend re-emerged, followed by a slight decrease after 2017.

Similarly, from 1990 to 2010, the APC in the ASYLDR for men in China displayed slight fluctuations, followed by a significant downward trend from 2010 to 2014, and a slight decrease thereafter.

Between 2001 and 2014, the APC in the ASYLDR for both men and women in China exhibited a downward trend. Specifically, for women, the ASYLDR displayed the following APC values: 2001–2004: − 0.935; 2004–2011: −0.222 (*P* < 0.05); and 2011–2014: −0.92. For men, the ASYLDR exhibited the following APC values: 2000–2010: −0.179 (*P* < 0.05) and 2010–2014: −1.657 (*P* < 0.05).

## Discussion

Our study, based on the GBD 2019 database, provides a comprehensive evaluation of the YLDs attributable to occupational noise-induced hearing loss in China over the past 30 years. Our analysis reveals the significant burden of hearing loss caused by occupational noise, with occupational noise accounting for 22.22% of the YLDs for hearing loss in 2019. From 1990 to 2019, the number of YLDs attributable to occupational noise-induced hearing loss showed an upward trend regardless of sex. However, when considering population growth, the APC in the ASYLDR attributable to occupational noise peaked in 2000 and then declined, reaching its lowest point in 2015. These findings have significant implications for policymakers, particularly in addressing the challenges posed by hearing loss.

Over the past few decades, the APC in the ASYLDR of hearing loss related to occupational noise increased from 1990 to 2000, followed by a downward trend, reaching its lowest point in 2015. From 2014 to 2017, there was a slight fluctuation in the data, attributable to China’s economic and social development during that period. From 1990 to 2000, China was undergoing rapid industrialization, with many people joining the workforce. However, the lack of awareness regarding the hazards of occupational noise, poor working conditions, and insufficient hearing protection led to a high incidence of occupational noise-induced hearing loss during this time. In 2002, the implementation of the “Law of the People’s Republic of China on the Prevention and Control of Occupational Diseases” marked the country’s formal recognition of the harm caused by occupational noise. Subsequently, the “Classification of Occupational Disease Hazard Factors” was released, which, for the first time, included noise-induced hearing loss in the list of occupational diseases. Following this, the attention to occupational noise-induced hearing loss gradually increased, workplace noise monitoring and management became more standardized, and workers’ awareness of occupational safety improved significantly, with more individuals actively using personal protective equipment (PPE). These developments may also be related to environmental reforms in the coal and thermal power sectors. Collectively, these factors contributed to the decline in the APC of occupational noise-related hearing loss ASYLDR after 2000. Therefore, although some fluctuations in the trend were observed, overall, the improvement in policies and public awareness has played a positive role in enhancing occupational safety.

Furthermore, we conducted a comparative analysis of the burden of occupational noise-related hearing loss in China by age and sex. Our study reveals that from 1990 to 2019, men consistently had higher YLD numbers and ASYLDR than women, aligning with most domestic and international reports [24]. This trend may be attributed to several factors. First, men are more likely to work in environments with poor working conditions, as evidenced by the fourth National Economic Census, which indicated that men constitute approximately 82.3% of the mining workforce [12]. Additionally, smoking, which is more prevalent among men in China, exposes smokers to different toxic substances in smoke that can synergistically affect hearing when combined with occupational noise [25–29]. Furthermore, women tend to have better hearing levels than men of the same age due to the protective effects of estrogen and its receptors on hearing [30, 31]. Moreover, women working in noisy environments often exhibit stronger self-protection awareness than men [32]. However, the rate of decline is less in women, which may be closely related to the increasing number of women working in noisy environments such as mining and manufacturing from 1990 to 2019.

Our results also indicate that with increasing age, both the number and rate of YLDs attributable to occupational noise-induced hearing loss generally exhibit an upward trend. Furthermore, the rate of decrease in YLDs among the older adults from 1990 to 2019 is slower than among young people. This phenomenon can be attributed to the progressive nature of occupational noise-induced hearing loss, where the extent of damage to the auditory system by noise is closely related to the duration of noise exposure, the nature of the noise, and its intensity. There is a clear dose-response correlation between workers’ hearing loss and noise exposur [33–35]. Workers aged 50 and above often have more than 10 years of work experience, leading to the accumulation of noise exposure in terms of duration and intensity. Additionally, older adults may also suffer from other health issues related to hearing loss, such as high blood pressure, and the use of medications such as aminoglycoside antibiotics, cisplatin, and loop diuretics, which are also risk factors for hearing loss [36–39].

From 1990 to 2010 and from 2010 to 2019, both Chinese women and men consistently exhibited a decrease in YLD numbers among young people. The rate of decrease in YLDs attributable to occupational noise among young people was notably rapid. Several factors may account for this phenomenon. First, young people typically have shorter exposure times to noise compared to their older counterparts [40]. Additionally, with the widespread availability of the internet and improved educational levels, young individuals have greater access to information and knowledge about protecting their hearing [41]. This enables them to realize the importance of hearing protection and take proactive measures to prevent and mitigate the impact of occupational noise. In contrast, some older adults may lack awareness of the importance of protecting their hearing or may not prioritize protective measures against occupational noise due to traditional beliefs or other reasons, resulting in a slower rate of decrease in hearing loss. Moreover, young individuals who experience symptoms such as tinnitus or hearing loss are more likely to seek prompt medical treatment and rehabilitation measures, thereby reducing the likelihood of occupational noise-induced hearing loss.

Therefore, it is imperative to intensify efforts to control occupational noise and mitigate its impact. The U.S. Department of Labor’s Occupational Safety and Health Administration has established legal limits for noise exposure in the workplace. The U.S. Department of Labor’s Occupational Safety and Health Administration’s permissible exposure limit is 90 dBA, with a time-weighted average for all workers of 8 h, while the National Institute for Occupational Safety and Health of the Centers for Disease Control and Prevention recommends an exposure limit of 85 dBA for an 8-h time-weighted average. Exposure at or above these levels is considered hazardous to hearing [42]. In China, “noise work” is defined as workplace noise intensity exceeding the “occupational exposure limit for harmful factors in the workplace,” which is an 8-h equivalent sound level (A-weighted) of ≥ 85 dBA. To comply with national regulations on exposure limits, employers must enhance the management and control of workplace noise to ensure that noise levels in all work environments do not exceed legal standards. This is crucial for safeguarding employees’ hearing health and ensuring work safety.

Multiple studies have revealed that wearing PPE, such as earplugs and earmuffs, can effectively reduce noise-induced hearing damage and lower the risk of hearing loss caused by occupational noise exposure [43–45]. Therefore, it is recommended that employers ensure workers consistently wear standard-compliant PPE and provide them with regular occupational health training to enhance their self-protection awareness and capabilities. Practical measures include: setting up noise monitoring points, monitoring noise levels in real-time, and adjusting protective equipment use based on noise intensity; implementing mandatory PPE regulations to ensure that all workers wear earplugs or earmuffs when noise exceeds safety limits; regularly replacing and maintaining protective equipment to ensure it remains in optimal condition. Additionally, supervisors should ensure compliance of the workers with the recommended protective measures toward maximizing the safeguarding of workers’ hearing.

In addition to wearing PPE, reducing noise at the source is one of the most direct and effective methods to minimize noise exposure and its associated hearing damage [13].

Noise monitoring and evaluation are also essential measures to mitigate the impact of noise on hearing [9]. Employers should establish noise monitoring points in high-noise areas, regularly measure noise levels, and ensure compliance with national noise exposure limits. If noise levels exceed the standard, specifically the 8-h equivalent continuous sound level (A-weighted) of ≥ 85 dB, effective control measures should be taken immediately, and the safety of the work environment should be reassessed. Regular noise assessments should include recording the duration of noise exposure to more accurately assess workers’ health risks and making adjustments to work schedules or shift rotations to reduce the impact of prolonged exposure on hearing. Moreover, the results of noise monitoring should be regularly communicated to workers, with a focus on raising their awareness of workplace noise levels and enhancing their desire to undertake protective measures.

As age increases, the detection rate of hearing loss attributable to occupational noise also increases [11]. Considering the serious aging population problem in China, employers should pay more attention to the hearing health of older adult workers. Additionally, the number of female workers in various industries in China is increasing, and the APC in the ASYLDR for women has not demonstrated a strong downward trend. This underscores the necessity of paying attention to the hearing health of female employees. As the population structure evolves, there is a pressing need to promptly adjust occupational health standards to more effectively protect workers’ hearing. Furthermore, previous research has consistently highlighted the mining and manufacturing industries as having elevated rates of hearing loss detection, and these industries persist today, with these traditional occupational disease hazards remaining significant. Moreover, as new professions, work types, and labor methods continue to emerge, they may introduce novel impacts on hearing health. Consequently, as the industrial structure evolves, a deeper understanding of the occupational health conditions in various industries becomes crucial, posing new challenges for occupational health standards.

## Conclusions

In conclusion, our study underscores the threat posed by occupational noise-induced hearing loss to public health in China, emphasizing the urgent need for action and intervention by health policymakers. Furthermore, our research identifies specific sex and age groups experiencing a higher burden of this disease, highlighting the importance of directing more attention and resources to these groups. The insights from this study are crucial for implementing effective measures to reduce occupational noise exposure in China. Notably, our study’s data is sourced from the GBD 2019 database, which, like any dataset, has certain limitations, including missing data in some areas that have not yet established a complete disease registration report.

## Data Availability

This study used publicly available deidentified data accessed from GBD (Global Burden of Disease) 2019, which are provided by the Institute for Health Metrics and Evaluation. https://vizhub.healthdata.org/gbd-results/.

## Abbreviations

AAPC: Average annual percentage change
APC: Annual percentage change
ASR: Age-standardized rate (per 100,000 population)
ASYLDR: Age-standardized YLD rate
DALY: Disability-adjusted life year
GBD: Global burden of disease
PPE: Personal protective equipment
UI: Uncertainty interval
YLDs: Years lived with disability
YLLs: Years of life lost
